# Decision-Making and Risk-Propensity Changes during and after the COVID-19 Pandemic Lockdown

**DOI:** 10.3390/brainsci13050793

**Published:** 2023-05-12

**Authors:** Pierpaolo Zivi, Stefano Sdoia, Valentina Alfonsi, Maurizio Gorgoni, Emanuela Mari, Alessandro Quaglieri, Luigi De Gennaro, Anna Maria Giannini, Fabio Ferlazzo

**Affiliations:** Department of Psychology, Sapienza University of Rome, Via dei Marsi 78, 00185 Rome, Italy; stefano.sdoia@uniroma1.it (S.S.);

**Keywords:** decision-making, cognition, stress, social isolation, COVID-19, lockdown

## Abstract

The imposition of lockdowns during the COVID-19 pandemic placed individuals under conditions of environmental stress, threatening individual and collective wellbeing. This study aimed to investigate the temporal effects of isolation and confinement during and after the Italian lockdown on decision-making, risk propensity, and cognitive control processes. The present study covered almost the entire Italian lockdown period (each week from the end of March to mid-May 2020), plus a follow-up measure (September 2020). At each time-point, respondents completed online behavioral tasks, which involved measuring risk-propensity (Balloon Analogue Risk Task), decision-making (Iowa Gambling Task), and cognitive flexibility (Category Switch Task). They also filled in questionnaires regarding subjective stress and anxiety. The main findings showed that the decision-making abilities of the respondents were affected as the confinement progressed. Furthermore, individuals who were more subjectively impacted by the lockdown/isolation experience exhibited impaired decision-making, especially during the lockdown. The results of the study highlight that prolonged confinement may affect human decision making, and help understand individuals’ misbehaviors during emergencies and develop effective countermeasures aimed at reducing the burden of the healthcare system.

## 1. Introduction

The outbreak and spreading of COVID-19 had a tremendous worldwide impact on societies, putting governments in a position where they had to make paramount decisions, especially regarding economics and healthcare. Many national governments implemented extraordinary measures to counteract the diffusion of COVID-19 and relieve the pressure on hospitals, especially Intensive Care Units. During the acute pandemic phase, millions of individuals were limited in their movements, with the closure of many national and regional borders. Moreover, in several countries, a national or territorial lockdown was imposed. Italy was among the first nations to undergo a national lockdown since it was the first Western country to fully deal with the COVID-19 pandemic. In Italy, the strict lockdown was imposed on 9 March 2020 and lasted until 4 May 2020.

Research has shown that the pandemic and some of the related countermeasures impacted individuals’ mental health [1]. Rapidly, an increase in the rates of suicides [2], sleep disturbances [3,4,5,6], eating disorders [7], anxiety and depressive symptoms [8,9], perceived stress [10], alcohol and substance abuse [11], and auto- and hetero-directed violence [12] emerged in the population, with some authors outlining a specific COVID Stress Syndrome [13]. Central features of COVID Stress Syndrome are the fear of contagion and the dangerousness of COVID-19, anxiety about the socioeconomic costs of COVID-19, xenophobic fears that foreigners can be spreaders, traumatic stress symptoms, compulsive checking, reassurance-seeking, avoidance, panic-buying, and difficulties coping during self-isolation.

The effects of stress on cognitive processes could underlie most of such features. Indeed, the literature reports that under stress, people exhibit reduced working memory resources [14,15,16] and narrowing of attention [17,18] and tend to use more habitual, simple, and low-effortful behavioral strategies at the expense of controlled and flexible ones [19,20,21]. Such effects might represent a major threat to individual and collective well-being during an emergency that requires both citizens and policymakers to take appropriate risks, carefully weigh action consequences, and make optimal decisions under uncertainty, i.e., when the outcomes of possible choices and the associated probabilities of occurrence are not known. According to the Somatic Marker Hypothesis [22,23], in such conditions, people must rely upon anticipated gut feelings based on previous experience when making decisions. However, when the ability to use such internal signals is compromised, individuals tend to rely upon immediate reward, increasing the rate of disadvantageous choices. Using the Iowa Gambling Task (IGT) [24], the analysis of anticipatory skin conductance responses as emotional markers of choices provided large support for the Somatic Marker Hypothesis [25]. In the IGT, participants must, for each of 100 trials, select one of four possible card decks differing in the gains and losses they provide. Two of the decks are high paying but result in losing points in the long run (i.e., are “disadvantageous”). Healthy participants typically learn to avoid them throughout the task and prefer sorting cards from the “advantageous” decks [26]. Research shows that stress affects decision-making performance on the IGT through making participants more prone to make risky and disadvantageous choices and affecting the learning trends [27,28]. Stress was also shown to affect performance on the IGT during the pandemic lockdown in Italy [29]. Indeed, the authors [29] found that in a one-shot study, perceived stress predicted task performance with age and sex differences, but only in the first 50 trials of the task (i.e., mostly under uncertainty [30]). Relatedly, in a longitudinal study conducted on Scottish nationals/residents during and after the pandemic lockdown [31], the IGT was administered together with other cognitive tasks (flanker, symbol-learning, and time production tasks) at 5 time points covering 13 weeks. In their study, [31] only the first time point represented a strict lockdown. Even though different tasks were performed in the population groups, researchers observed a constant improvement in all the tasks. As the authors observed, such an improvement can be attributed to the progressive easing of restrictions or practice effects.

Most studies investigating decision-making under stress conditions focused on acute effects in laboratory settings, reporting, among other outcomes, altered feedback processing using gambling tasks [32]. These tasks, such as the IGT or the BART (Balloon Analogue Risk Task [33,34]), provide an indication of risk-seeking behavior in uncertain conditions. For each trial of the BART, participants must inflate a balloon to win points. However, the balloon might explode during a given trial, with a random probability causing the loss of the points potentially earned during that trial. Thus, the BART score is positively associated with individual risk propensity. Acute stress was found to affect risk-seeking behavior during the BART with gender differences [34,35,36]. The stress-related change in risk-seeking and decision-making largely depends upon individual differences and the situation with which the individuals are dealing [32,34,35,36,37,38,39,40,41]. Indeed, it was highlighted [32] that the IGT and BART importantly differ in the risk-reward contingencies they subtend. On one hand, a risk-seeking behavior in the IGT leads participants to preferentially sort from high-paying decks. However, those decks would lead participants to lose points in the long run and perform disadvantageously. On the other hand, a risk-seeking behavior in the BART would lead participants to inflate the balloon with a high number of pumps in each trial. Thus, such behavior would lead participants to win more in the long run and perform advantageously. Therefore, the complementary use of the IGT and the BART to inspect stress-related effects on decision-making would help to distinguish between a pure risk-seeking hypothesis (i.e., stress increases overall risk-seeking) and a suboptimal adjustment of the decision-making strategy to perform advantageously.

Social isolation might represent a prolonged stress condition [42]. Prolonged stress is showed to modulate risky behavior and affect decision-making, even though studies in this regard are limited (e.g., [27,43,44,45,46]). Lockdowns and social distancing policies compelled individuals to live in restricted and confined spaces. Forced cohabitation during the lockdown periods was shown to affect perceived stress and coping strategies [47]. Restricted space and crowded environments were shown to increase skin conductance levels [48] and be positively correlated with cortisol concentrations [49]. Studies show that long-term living in an isolated and confined environment (ICE) might have negative psychological outcomes. ICE environments [50] present a variety of environmental factors challenging human adaptation [51,52]. ICEs (e.g., polar stations, submarines, caves, bed-rest studies, isolation facilities) are frequently used as analogs of space missions since they share several features with the spaceflight itself. Monotony, reduced stimulation, and confinement represent psycho-environmental sources of stress in these contexts [53,54], requiring the implementation of valid countermeasures, such as physical exercise [55,56,57]. Most prior research in this area addressed the impact of prolonged confinement on affective processes [58,59,60,61], group dynamics and psychosocial issues [58,62,63], and cognitive and psycho-physiological processes [64,65,66]. More generally, social isolation and feelings of loneliness are shown to reduce cognitive control processes’ efficiency [67] and overall cognitive performance [42]. Furthermore, the effect of social isolation is particularly prominent for older individuals, who are more at risk of withdrawing from social life and may suffer the impact of social isolation on global cognitive functioning, including executive functions and memory [68]. Therefore, it is important to account for individuals’ age when considering the psychological and cognitive effects of the exceptional condition represented by the pandemic lockdown.

Within this picture, the present work reports the cognitive part of a broader study conducted during the COVID-related lockdown in Italy [3,4,10]. Specifically, it aimed to add knowledge on the potential cumulative effects of prolonged isolation due to the pandemic lockdown on cognitive processes, especially decision-making. Through administering two standard decision-making tasks, namely the IGT and the BART, this study sought to concurrently investigate decision-making under uncertainty and risk propensity through hypothesizing different effects. Indeed, if the lockdown-related isolation and stress affected risk-seeking, an increase in the BART score and a decrease in the overall IGT net score (i.e., more disadvantageous choices) would be observed. Alternatively, a reduction in both the BART and the IGT scores would be observed if the ability to learn and use reward-risk contingencies were compromised. Moreover, a measure of task-switching to investigate control processes was also included. Indeed, if the effects of social isolation and stress on decision-making were associated with modulations regarding the efficiency of control processes, a concurrent reduction in task-switching abilities would be observed.

## 2. Materials and Methods

### 2.1. Participants

**Figure 1:**
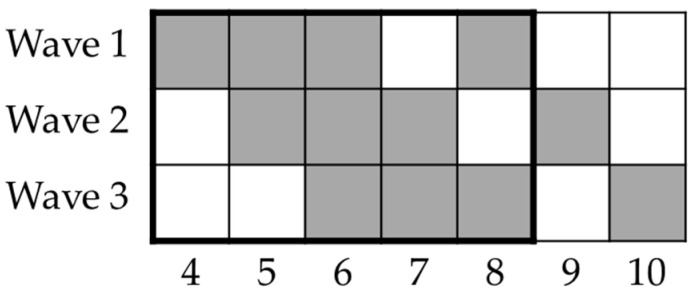
Timeline of study’s lockdown phase. Columns represent weeks since lockdown onset (9 March 2020); rows represent waves of participants enrolled. Black rectangle depicts experimental weeks within lockdown period in Italy (until 4 May 2020). Participants were asked to perform tasks on weeks depicted using grey squares (i.e., every week for three weeks and then two weeks after this period).

This study was designed when the first Italian lockdown had recently been imposed. Participants were enrolled online through social media and university networks, and were prompted to respond every week for three consecutive weeks and then two weeks later; however, they were not implored to respond in other weeks. With the aim of disentangling the time course spent confined at home and the time course spent participating in the study, participants were enrolled in three moments that were one week apart (see Figure 1 for details about the timeline of the lockdown phase of the study), determining three waves of participants. The first wave of respondents started the study on 28 March 2020. An additional time-point was designed four months after the end of the lockdown to include an overall follow-up measure; this stage took place in September 2020. For the whole lockdown phase, up to 477 different participants (146 males and 331 females; M age 33.26) were included in the analyses (see Methods and Results). However, up to 167 participants (50 males and 117 females, M age 33.96) were included in the follow-up analysis (see Methods and Results). Further information about the sample is provided in Table 1 and Section 3. The study was approved by the Institutional Review Board of the Department of Psychology at the Sapienza University of Rome and conducted in accordance with its policies and the Declaration of Helsinki. All participants provided informed consent.

### 2.2. Cognitive Tasks

The three cognitive tasks administered were implemented on the Inquisit platform (www.millisecond.com), modifying the parameters of the library scripts. The Iowa Gambling Task (IGT) [24], Balloon Analogue Risk Task (BART) [33], and Category Switch Task (CST) [69,70,71] were administered to all participants (see Appendix A for details).

### 2.3. Questionnaires

Due to their potential to affect decision-making and executive functions, stress and anxiety indices were included in the study as predictors of behavioral performance. The rationale underlying this choice was that since those dimensions were central in the COVID-related stress symptomatology, it was in the interest of this study to separate their effect on behavioral performance as much as possible from the pure effect of lockdown-related prolonged social isolation. An overview of the results of the subjective measure were reported elsewhere [3,4,10]. Details on the subjective measures used, namely the Impact of Event Scale (IES) [72], the State-Trait Anxiety Inventory (STAI) [73], and the Perceived Stress Scale (PSS) [74], are reported in Appendix A.

### 2.4. Procedure

Participants entered the research website at each experimental time-point and launched the Inquisit link for the cognitive tasks. The order of the tasks was fixed, while the order of the questionnaires varied depending on time-points. The BART was the first task completed, followed by the IGT, CST, and Psychomotor Vigilance Task (findings on the final task will not be discussed in the present study). Respondents provided informed consent at their first experimental session through Qualtrics and used the same personal code for all the sessions in which they participated. They were required to complete all the measures within the same experimental week.

### 2.5. Statistical Analyses

Before the analyses, data were cross-checked to match performance, subjective, and demographic indices for each observation. The observation was excluded if a match between performance and subjective/demographic indices was not possible. Only the first response was considered if the same participant responded more than once during the same experimental week. Only data from participants who stated that they were located in Italian territory during the study were analyzed. Since only completed tasks were considered, sample sizes differed among the three tasks, and there were no missing data. Two different sets of data analyses were planned. A first analysis was run on the consecutive time-points during the lockdown to inspect the presence of a possible trend in the cognitive performance throughout the isolation/lockdown period. Since respondents were not consistent with the longitudinal design, linear mixed-effects models were implemented separately for each task outcome (IGT score, BART score, CST switch cost, and CST N-2 repetition cost). The main models included Week (and its polynomial terms) and a variable indicating the number of previous responses provided in the study (Previous Sessions) to control for practice effects. For the IGT model, an additional Block variable (and polynomial terms) comprised the five blocks of trials of the task. For each task outcome, the best models were estimated considering socio-demographic (age, sex, and a dummy variable indicating whether participants happened to leave home during the day) and subjective data (PSS, STAI-Y1). Participants’ IDs were included as a random intercept (see Appendix A for further details). Due to the low number of observations in weeks 9 and 10, level 9 of the Week factor resulted from the merger of the two weeks (both weeks followed the end of the lockdown, see Figure 1). Thus, this study had six consecutive time-points (from 28 March to 18 May 2020). The sample sizes for each time-point in the lockdown analyses are reported in Table 1. The second analysis was run to investigate the differences between the lockdown and follow-up measurements exerted via the subjective impact of the lockdown experienced by respondents. This analysis was run on each task outcome using separate mixed ANCOVA designs using Session (Lockdown and Follow-Up) as a within and IES group (low or high, based upon the median IES score) as a between-subjects factor. We also included Age and the STAI-Y2 score as continuous covariates (see Appendix A for further details).

## 3. Results

### 3.1. Iowa Gambling Task

The analyses for the IGT were conducted using 1256 observations (470 different participants; 143 males and 327 females; M age 32.98, SD 14.31; M observations per participant 2.67, SD 1.32). The best-fitting model was that with Week, Previous Sessions, Block, and Age as predictors, without the interaction between Week and Block. Age, Previous Sessions, and the linear effect of Block were significant, whereas the effect of Week was not significant (see Table A1). However, the linear effect of the Week factor became significant with the bootstrap (Table A1), revealing a decreasing trend. The effect of Block was due to the typical learning observed throughout the task blocks [26]. The effect of Previous Sessions described an increase in the score as the number of previous performed sessions increased, probably due to a practice effect, whereas the Age effect indicated that older adults chose less advantageously than younger adults.

**Figure 2:**
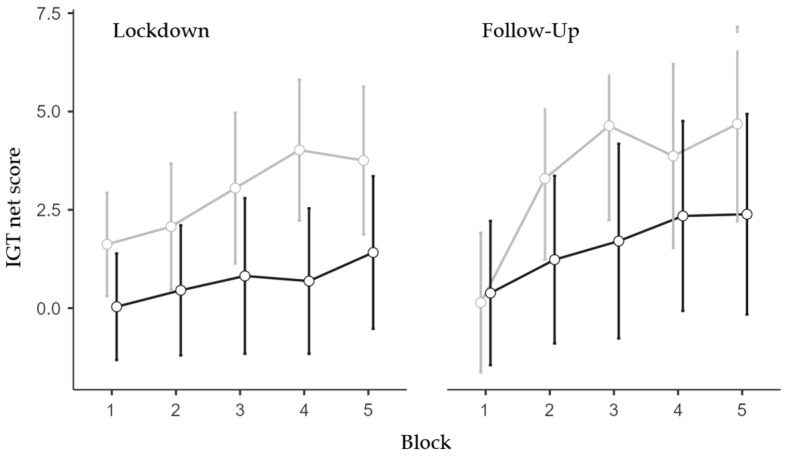
IGT net score for IES Group, Time-Point, and Block. Covariate means: Age = 34.05, Trait Anxiety = 45.15. Grey line represents low IES group; black line represents the high IES group. Vertical bars denote 95% confidence intervals.

The only significant effect found in the follow-up ANOVA (N = 163; 113 females and 50 males; 84 low and 79 high IES) was the main effect of the IES Group (F1,159 = 4.08, *p* = 0.045, η^2^*p* = 0.025). Specifically, the high-IES group exhibited less advantageous choices than the low-IES group (Figure 2). All other effects were non-significant (see Appendix B).

Trend analyses were conducted on the performance scores throughout the five blocks separately for the IES group and Session to further confirm such an effect. The low IES group exhibited a significant linear trend in the lockdown session (*p* < 0.01, *p* = 0.47, and *p* = 0.23, for the linear, quadratic, and cubic trends, respectively) and significant linear (*p* < 0.001) and quadratic (*p* = 0.026) trends (*p* = 0.14 for the cubic trend) in the follow-up session. Differently, the high IES group did not exhibit significant trends either in the lockdown session (*p* = 0.18, *p* = 0.95, and *p* = 0.55 for the linear, quadratic, and cubic trends, respectively) or at follow-up (*p* = 0.06, *p* = 0.64, and *p* = 0.96, for the linear, quadratic, and cubic trends, respectively).

Altogether, the IGT data analyses showed that individuals who were more impacted as a result of the lockdown/isolation chose less advantageously than less impacted individuals. This reduced ability was more evident during the confinement period but protracted during the following months (Figure 2).

### 3.2. Balloon Analogue Risk Task

**Figure 3:**
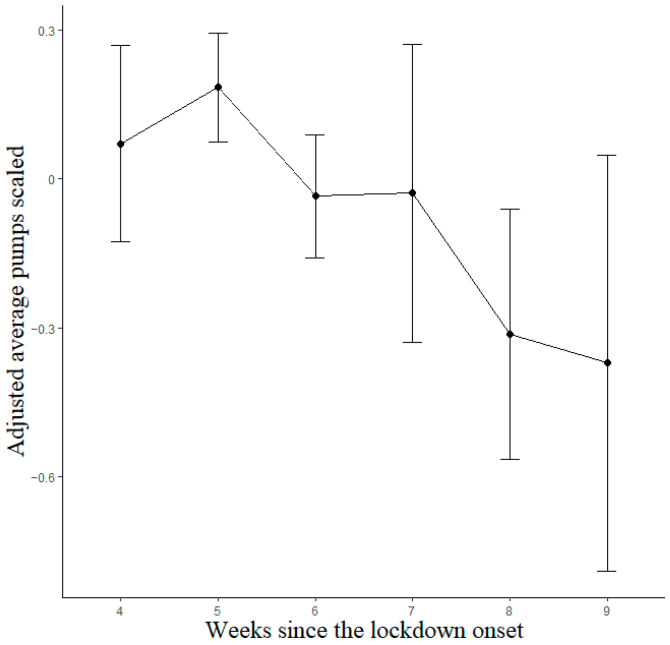
Estimated marginal means for BART lockdown model. Vertical bars denote 95% confidence intervals.

Observations with scores equal to 0, indicating that participants did not perform the task at all, were excluded from the analyses. A total of 1265 observations (477 different participants; 146 males and 331 females; M age 33.26, SD 14.47; M observations per participant 2.65, SD 1.32) were analyzed through linear mixed models for the lockdown data. The best-fitting model was that with Week and Previous Sessions as predictors. The Previous Sessions predictor and linear term of the Week factor reached statistical significance; after bootstrapping, the significant effects were maintained (see Table A1). Importantly, the two estimates had the opposite sign, indicating opposite trends. Participants exhibited decreasing BART scores as the lockdown progressed (Figure 3), with Bonferroni-corrected post hoc contrasts showing significant differences between weeks 5 and 8 (*p* = 0.014) and between weeks 6 and 8 (*p* = 0.046), whereas all other contrasts were non-significant (*p* > 0.05).

**Figure 4:**
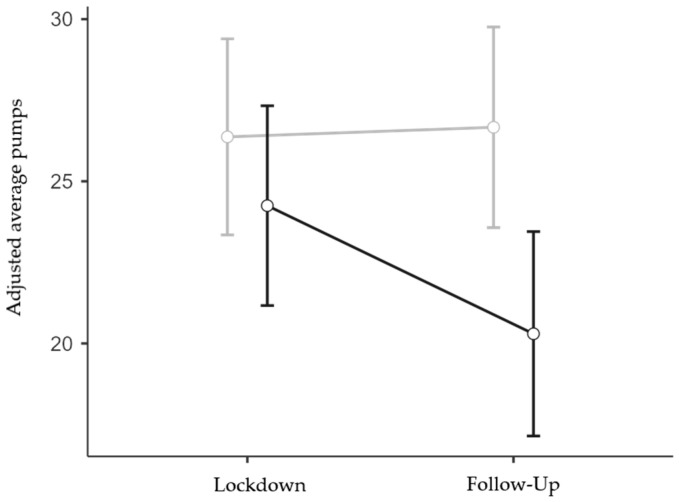
Interaction effect between IES Group and Session on BART adjusted average pumps. Covariate means: Age = 33.96, Trait Anxiety = 45.40. Grey line represents low IES group; black line represents high IES group. Bars denote 95% confidence intervals.

Interestingly, it was found in the follow-up ANOVA (N = 167; 117 females and 50 males; 85 low and 82 high IES) that the more impacted respondents exhibited more risk-averse behavior than the less impacted ones, but only at the follow-up stage (IES group x Session interaction effect: F1,163 = 4.08, *p* = 0.045, η^2^*p* = 0.024; Figure 4). The interaction between Session and Age was also significant (F1,163 = 5.67, *p* = 0.018, η^2^*p* = 0.034), but the interaction between Session and TA was not significant (F1,163 < 1, *p* = 0.418, η^2^*p* < 0.01). Finally, the main effect of IES Group was significant (F1,163 = 4.57, *p* = 0.034, η^2^*p* = 0.027), while the main effect of Session (F1,163 = 3.53, *p* = 0.062, η^2^*p* = 0.021) and the effects of the two covariates were not significant (F1,163 < 1, *p* = 0.381, η^2^*p* = < 0.01 and F1,163 < 1, *p* = 0.962, η^2^*p* = < 0.01 for Age and TA, respectively). Bonferroni-corrected post hoc contrasts confirmed that the reduction in risk propensity observed in the High IES group at the follow-up stage was statistically significant when compared to all the other Group x Session conditions (*p* < 0.05 for all three comparisons), whereas the other comparisons were not statistically significant (*p* = 1).

Taken together, the results from the BART revealed a reduced risk propensity as the lockdown/isolation period approached its end (Figure 3). Such a decrease was more evident for individuals more impacted by the lockdown/isolation experience, for whom it was protracted for four months (Figure 4).

### 3.3. Category Switch Task

A prior ANOVA on the lockdown dataset was conducted to search for a reliable switch cost. One-way ANOVAs on RTs were conducted using Trial Type (Repetition vs. Switch Trial) as a within-subject factor. A significant effect from Trial Type was found (F1,1261 = 1748.31, *p* < 0.001, η^2^*p* = 0.58), indicating that respondents were slower in responding to switch than repetition trials (RT means were 1037.03 ms and 1354.58 ms for repetitions and switches, respectively). The same ANOVAs on RTs for ABA and BBA sequences of trials were conducted to ensure that an N-2 repetition cost was also reliably present. The ANOVA showed a significant main effect from Sequence (F1,1261 = 183.784, *p* < 0.001, η^2^*p* = 0.13), indicating that participants were slower to perform a recently abandoned task than a less recently abandoned task after a switch (RT means were 1394.31 and 1317.49 ms for N-2 repetition and N-2 switches, respectively).

For the construction of linear mixed models for the lockdown data, observations with accuracy lower than 3 SD from the grand mean were excluded (91.9%, SD 10.1%), resulting in 1219 observations (462 different participants; 137 males and 325 females; M age 32.87, SD 14.28; M observations per participant 2.64, SD 1.29). Estimates obtained using mixed models for both switch and N-2 repetition costs are reported in Table A2. As regards Switch Cost, the best model included Week, Previous Sessions, and Age as predictors. A practice effect was highlighted using the significant Previous Sessions factor, indicating that Switch Cost progressively reduced as respondents participated in the study. The effect of Age was also significant, with older adults exhibiting higher costs. The cubic term of Week was also significant, with this effect being maintained after bootstrapping (Table A2). Regarding N-2 repetition cost, the selected model included only Week and Previous Sessions as predictors. Even in this case, a significant effect of Previous Sessions showed a reduction in the cost, which is in line with a practice effect. The bootstrapping procedure revealed a significant linear component of the Week factor, indicating that N-2 repetition costs increased as the lockdown progressed. However, for both outcomes, pairwise contrasts among the levels of the Week factor did not show any significant difference (*p* > 0.05 for all).

Two separate follow-up analyses were performed for the switch and N-2 repetition cost. Participants with less than 3 SD from the grand-mean accuracy were excluded (N = 161; 112 females and 49 males; 81 low and 80 high IES; covariate means: 33.75 for age and 45.32 for TA). The follow-up analysis on the switch cost revealed only a significant effect from the Age covariate (F1,157 = 11.03, *p* = 0.001, η^2^*p* = 0.066). All other effects were non-significant (see Appendix B). In the low-IES group, estimated marginal means for the switch-cost were 265.82 ms (95% CI 221.50–310.14) and 236.1 ms (95% CI 195.70–276.49) for the lockdown and follow-up stages, respectively; in the high-IES group, the means were 282.68 ms (95% CI 238.08–327.28) and 235.81 ms (95% CI 195.16–276.46) for the lockdown and the follow-up stages, respectively.

The follow-up analysis on the N-2 repetition cost revealed no significant effects at all (see Appendix B). In the low-IES group, estimated marginal means for the N-2 repetition-cost were 57.18 ms (95% CI 29.12–85.24) and 40.06 ms (95% CI 5.96–74.16) for the lockdown and the follow-up stages, respectively; in the high-IES group, the means were 78.93 ms (95% CI 50.69–107.17) and 53.99 ms (95% CI 19.67–88.31) for the lockdown and the follow-up stages, respectively.

Overall, typical practice effects were found throughout the study for the CST. Moreover, confinement differently affected these different measures of task-switching. Moreover, N-2 repetition cost, as a measure of backward inhibition [71], showed an increasing trend, but no significant differences were found between the sessions. It is worth noting that this was the only cognitive task in which the dependent variable was measured through reaction times. Since we performed remote research, the scarce methodological control over the respondents’ set-ups may have concealed any subtle effect.

## 4. Discussion

While most investigations on the time-related negative impact of the lockdown/isolation on psychological processes used subjective measures and surveys, only a few pieces of evidence collected through standard behavioral measures exist [29,31].

In summary, the results of the present study highlight that the respondents exhibited time-dependent changes in decision-making abilities. Globally, controlling for repeated administration, BART and IGT scores decreased as the confinement progressed. Moreover, a within-subjects comparison between lockdown and follow-up sessions showed that the individuals who were more impacted by the experience of the lockdown/isolation (assessed through the Impact of Event Scale) chose less advantageously in the IGT (lower scores) during both the phases, but particularly during the lockdown, in comparison with the less impacted respondents. Relatedly, only the highly impacted individuals exhibited reduced risk-seeking (lower scores) in the BART four months after the lockdown, indicating that the global effect observed was prolonged in individuals whose experience felt more stressful. Changes in risk preferences were investigated during the pandemic, but results are still not conclusive and might depend upon specific conditions [75,76]. Unfortunately, the literature lacks useful studies for comparison. In general, studies reporting the effects of stress exposure on decision-making abilities reported a shift toward the use of habitual strategies [20,77], an increase in the rate of disadvantageous and risky choices [28,34,36,39,40,41], and an alteration of feedback sensitivity [78,79,80]. Stress administration was shown to affect decision-making under uncertainty using the IGT [28,41,81,82].

In terms of risk-propensity behavior, the two results obtained appear to be contradictory at first glance. Low BART scores underlie risk-avoidant behaviors, while low IGT scores underlie risk-seeking behaviors. However, the two tasks differ for the subtended reward–risk contingencies [32,40]: a riskier approach results in lower gains in the IGT (disadvantageous) and higher gains in the BART (advantageous). Thus, in this study, more impacted individuals exhibited more disadvantageous decision-making behaviors in both tasks, highlighting that changes were not the results of an overall increase in risk-seeking. Instead, the observed pattern presumably regards processes involved in feedback learning.

The social isolation due to the COVID-19 lockdown represents a prolonged stressful condition. Hauche et al. [83] showed that individuals exhibited higher levels of cortisol (i.e., a hormonal marker of the stress response) during the lockdown compared to a non-lockdown period. The activity of the HPA axis (hypothalamic–pituitary–adrenal) and consequent release of cortisol were stimulated through stressful experiences, causing changes in several brain areas, such as the amygdala and the prefrontal cortex [84]. These areas were shown to be largely involved in decision-making under uncertainty, specifically in feedback processing and learning [24,32]. For instance, the literature suggests that during decision-making the orbitofrontal cortex and ventromedial prefrontal cortex are implicated in the processes underlying the coupling between the affective and cognitive components of choices [85,86]. Accordingly, stress can impair the connections between the prefrontal cortex (involved in high-level cognitive functioning) and the amygdala [87]. The Somatic Marker Hypothesis [23] suggests that during the decisional process, the immediate and delayed prospects of the available options are driven via subcortical and cortical mechanisms, possibly exerting conflicts among somatic responses produced via the available options [88]. Thus, dissociations between such “impulsive” and “reflective” networks can be observed when considering the effects of stress on decision-making processes.

Relatedly, it was elsewhere reported that respondents experienced a period of emotional flatness during the lockdown [10], which was discussed in terms of an adaptive coping strategy useful in switching off from a stressful experience, and already observed in individuals who have been confined for a long time [61]. Such a detachment from emotional experience was arguably the core mechanism leading to the observed disadvantageous behavior.

Long-term confinement, restricted environment, reduced stimulation, monotony, forced cohabitation with unvarying individuals, separation from friends and/or relatives, and lack of privacy are among the major psychosocial threats that individuals experience in isolated, confined, and extreme environments [50]. As processive stressors, confinement-related stressors do not necessarily present an immediate threat to the organism but can be recognized, interpreted, and anticipated. Structural social life aspects may represent protective factors for the mental health of individuals experiencing emergencies [89]. These aspects should be taken into consideration for interventions during emergencies and in prevention campaigns. Even in an exceptional and weakly controlled condition (e.g., the absence of a pre-lockdown measure), evidence from behavioral science might be precious in understanding individuals’ conduct during the crisis, providing useful information for the prevention and implementation of effective countermeasures [90].

## 5. Conclusions

Findings suggest that individual decision-making and risk propensity can be affected by the lockdown experience. This work highlights the possible mechanisms involved in risky, counter-productive behaviors resulting from home confinement during an emergency, which might be highly dangerous for individuals and communities.

## Figures and Tables

**Table 1:** Sample sizes used in linear mixed models of lockdown effects. Week column represents weeks since lockdown onset. Weeks 9 and 10 were grouped in Week 9 level.

Week	BART	IGT	CST
4	N = 408	N = 402	N = 386
	124 m, 284 f	122 m, 280 f	114 m, 272 f
	Age M 32.57 SD 14.33	Age M 32.42 SD 14.24	Age M 32.29 SD 14.20
5	N = 314	N = 310	N = 301
	96 m, 218 f	94 m, 216 f	89 m, 212 f
	Age M 33.03 SD 14.67	Age M 33.13 SD 14.75	Age M 32.73 SD 14.57
6	N = 292	N = 292	N = 286
	91 m, 201 f	91 m, 201 f	87 m, 199 f
	Age M 34.20 SD 15.17	Age M 34.10 SD 15.11	Age M 34.04 SD 15.07
7	N = 27	N = 27	N = 26
	7 m, 20 f	7 m, 20 f	6 m, 20 f
	Age M 39.11 SD 15.57	Age M 39.11 SD 15.57	Age M 39.69 SD 15.58
8	N = 200	N = 200	N = 195
	62 m, 138 f	63 m, 137 f	58 m, 137 f
	Age M 33.32 SD 14.77	Age M 33.17 SD 14.75	Age M 32.92 SD 14.62
9	N = 24	N = 25	N = 25
	4 m, 20 f	4 m, 21 f	4 m, 21 f
	Age M 31.42 SD 15.15	Age M 31.00 SD 14.98	Age M 31.00 SD 14.98

## Data Availability

The data that support the findings of this study are available on request from the corresponding author. The data are not publicly available due to restrictions, e.g., containing information that could compromise the privacy of research participants.

## Appendix A. Materials and Methods

### Appendix A.1. Iowa Gambling Task

The Iowa Gambling Task (IGT) [24] is a behavioral task aimed at assessing decision-making under uncertainty. For each of the 100 trials, respondents must choose one card from one of the four decks. Each card allows some amount of money to be won or lost. Unknown to participants, the four decks differ in the frequency and magnitude of wins and losses. Losses are more frequent in two decks (A and C) than in the other two decks (B and D). However, A and B are high-paying decks and present a higher magnitude of losses. The opposite is true for the low-paying decks (C and D), which have lower losses but pay more in the long run. Thus, decks A and B are “disadvantageous”, whereas decks C and D are “advantageous”. Gains and losses were the same as in the Bechara’s standard paradigm [24]. The standard IGT score was calculated as the number of advantageous choices minus the number of disadvantageous choices [(C + D) − (A + B)] in each of the five blocks of 20 trials. The literature showed [26] that healthy participants usually learn the identity of the two advantageous decks throughout the task and prefer to choose between them in the last 2 or 3 blocks. In the present study, participants started with 2000 virtual euros and were told to win as much as possible. Given the experimental design, to control for learning effects throughout the study period, the order of the decks on the screen was randomly manipulated into four possible combinations and their reward through multiplying gains and losses by a factor of 1, 2, or 3.

### Appendix A.2. Balloon Analogue Risk Task

The Balloon Analogue Risk Task (BART) [33] is an uncertainty-based decision-making task aimed at assessing risk propensity. In this task, respondents are presented with one of 30 balloons in each trial. For each balloon, respondents must inflate the balloon as much as they want, either temporarily gaining money for each pump or permanently collecting their winnings and skipping to the next balloon. The payoff for each pump was set to 0.05 points. Participants are told that the balloon may explode after any pump, making them lose the potential winnings for that balloon. The maximum number of possible pumps for each balloon is 128; thus, the probability that a balloon might explode after the first pump is 1/128. This probability increases after each pump. Participants are unaware of the maximum number of pumps or the explosion probabilities. They know that a balloon might explode after the first pump or that a balloon might fill up the entire screen without exploding. Participants are told that their goal is to earn as much as possible. As a standard measure, the adjusted average number of pumps was calculated as the average number of pumps for the unexploded balloons.

### Appendix A.3. Category Switch Task

Participants are presented with a word in each trial in the Category Switch Task (CST) [69,70]. According to one of two categorization rules, they must respond as fast and accurately as possible. The “living” task is prompted using a heart-shaped cue and requires participants to categorize words as living or non-living objects; the “size” task is prompted using an arrow-cross cue and requires participants to categorize words based on their size (bigger or smaller) relative to a basketball. For each trial (except the very first one), participants can be asked to perform the same task performed in the previous trial (i.e., repetition trial, “living-living” or “size-size”, AA or BB) or the other task (i.e., switch trial, “living-size” or “size-living”, AB or BA). Half of the trials are switch trials, while half are repetitions, with both trials being presented randomly. Congruent and incongruent (same/different responses required by the tasks on two successive trials) trials are also equally balanced. Participants are required to respond on their keyboard using their left and right index fingers on the ‘E’ and ‘I’ keys, respectively. Responses were randomly assigned to the key mappings. The task consisted of a practice and an experimental phase. During the practice phase, participants performed 16 trials for the “living” task and 16 trials for the “size” task in balanced order across participants. Next, 16 practice trials were presented wherein both the tasks were presented as in the experimental phase. Additional practice trials were presented if participants’ accuracy was lower than 80% during the practice. The experimental phase consisted of 64 trials. The intertrial interval was set at 500 ms for correct responses and 1500 ms for errors. The cue (heart or arrow) and target (the word) were presented simultaneously in each trial. Average RTs in the experimental phase were separately computed for repetition and switch trials for each participant. The switch-cost for each participant was calculated as the mean RT for switch trials minus the mean RT for repetition trials. Moreover, the N-2 repetition cost (i.e., the persistent interference of previous task sets in current performance [71]) was calculated as the difference between RTs observed on the third task of ABA or BAB triplets (“living”–“size”–“living” or “size”–“living”–“size”) and on BBA or AAB triplets (“size”–“size”–“living” or “living”–“living”–“size”). RTs smaller/larger than two standard deviations below/above the individual mean at each time-point were considered outliers and excluded from the analyses. Error trials were discarded for the computation of RTs.

### Appendix A.4. Impact of Event Scale

The 15-item Italian version of the Impact of Event Scale (IES) [72] was administered to respondents at the follow-up session. Respondents rated each item using a four-point scale. The IES score (the sum of the score on each item) measures to what extent the lockdown experience has impacted individuals, with high scores indicating a stronger impact, tapping event-related intrusive and avoidant experiences.

### Appendix A.5. State-Trait Anxiety Inventory

The Italian version of the State-Trait Anxiety Inventory (STAI) [73] was administered to evaluate individuals’ state (STAI-Y1, on each time-point) and trait (STAI-Y2, at the first and follow-up sessions) anxiety. The two scales each consisted of 20 four-point items, where higher scores indicated higher anxiety levels.

### Appendix A.6. Perceived Stress Scale

The Italian version of the Perceived Stress Scale (PSS) [74] was administered at each time-point to investigate the subjective feelings of stress during the whole experimental period. The PSS is a five-point scale (0–4) consisting of 10 items. Higher scores indicate a greater subjective experience of stress.

### Appendix A.7. Statistical Analyses

As regards the lockdown analysis, linear mixed-effects models were implemented separately for each task using the *lme4* package [91] within the *R* environment. The main models included two time-related variables. The factor “Week” (and its polynomial terms) indicates the ordinal number of the experimental week since the lockdown onset (4, 5, 6, 7, 8, and 9). Instead, “Previous Sessions” represents a variable describing how many sessions in which the participant had already participated (0 = first session), allowing us to control for possible practice effects. For instance, for data collected during week 8 from one participant who already responded during weeks 4 and 6, the value of Previous Sessions is 2.

Firstly, the best linear models were estimated for each task using performance outcome (BART adjusted average pumps; IGT net scores; CST switch cost and N-2 repetition cost) entering stepwise subjective (PSS, STAI-Y1), socio-demographic (sex and age plus a dummy variable indicating whether respondents happened to go out during the day), and temporal predictors (Week and Previous Sessions). A Block variable (blocks of trials: 1, 2, 3, 4, and 5) and its polynomial terms were also included in the IGT model. All the quantitative variables, except for Week and Block, were centered around their respective mean and scaled. Since the focus of the study was the temporal effect of the lockdown regarding cognitive performance, the interaction terms were not included in the models (the only exception is the Week by Block interaction in the IGT model). A random intercept for participants’ ID was then entered, and the selection process proceeded backwards, eliminating at each step the non-temporal predictor with the highest p-value. The best model was chosen according to AIC and BIC. In case of disagreement, the more parsimonious model was selected. For the post hoc contrasts, which were run using the emmeans package for *R* [92], the Satterthwaite approximation was applied to the degrees of freedom, and the *p*-values were Bonferroni-adjusted. In order to control for the unbalanced sample sizes across the time-points, sampling distributions of the estimates were constructed using a bootstrapping approach (N = 1000) after selecting the final model.

The second analysis was conducted to investigate whether the individual behavioral performance changed during the follow-up period, according to the impact subjectively experienced during the confinement. To ensure that the lockdown measurement was not influenced through possible effects due to the first session or proximal end of the lockdown period, among the participants who responded at the follow-up stage, only participants who responded on Week 1 and at least on one between Week 2 and Week 3 were retained. As a lockdown measurement, the score on Week 2 or Week 3 was used; the mean between the two scores was used if participants responded on both weeks. Participants were then split into two groups (Low and High IES) using the median of the IES score obtained during the follow-up stage. Thus, separate mixed ANCOVAs were run for the tasks’ outcomes, using Session (2 levels: Lockdown and Follow-Up) as a within-subjects factor and IES group (2 levels: Low IES and High IES) as a between-subjects factor. An additional repeated-measures Block factor (5 levels: 1, 2, 3, 4, and 5) was included in the analysis of the IGT data. In case of significant violation of sphericity, Greenhouse–Geisser correction was applied. Since evidence suggests that Trait Anxiety (TA) affects decision-making under uncertainty [93], the STAI-Y2 score obtained on week 1 was included as a continuous covariate. Age was also included as a covariate to control for possible confounding effects due to age differences in the sample. For post hoc contrasts, *p*-values were Bonferroni-corrected.

## Appendix B. Results

### Appendix B.1. Iowa Gambling Task

In the follow-up ANOVA, besides the only significant effect of the IES group, all the other effects were non-significant (F1,159 = 1.834, *p* = 0.178, η^2^*p* = 0.011; F3.57,567.661, F = 1.235, *p* = 0.295, η^2^*p* < 0.01; F1,159 = 1.062, *p* = 0.304, η^2^*p* < 0.01; F1,159 < 1, *p* = 0.648, η^2^*p* < 0.01 for the main effects of Session, Block, Age, and TA, respectively; F1,159 < 1, *p* = 0.828, η^2^*p* < 0.01; F1,159 = 1.982, *p* = 0.161, η^2^*p* = 0.012; F1,159 < 1, *p* = 0.699, η^2^*p* < 0.01 for the interaction of Session with Age, TA, and IES group, respectively; F3.57,567.661 < 1, *p* = 0.493, η^2^*p* < 0.01; F3.57,567.661 = 1.48, *p* = 0.212, η^2^*p* < 0.01; F3.57,567.661 = 1.11, *p* = 0.349, η^2^*p* < 0.01; for the interactions of Block with Age, TA, and IES group, respectively; F4,636 = 1.424, *p* = 0.224, η^2^*p* < 0.01; F4,636 < 1, *p* = 0.435, η^2^*p* < 0.01; F4,636 < 1, *p* = 0.467, η^2^*p* < 0.01; F4,636 < 1, *p* = 0.477, η^2^*p* < 0.01; and for the interactions between Block and Session, Block and Session and Age, Block and Session and TA, and Block and Session and IES group, respectively).

### Appendix B.2. Category Switch Task

In the follow-up ANOVA on the switch cost, besides the only significant effect of the Age covariate, non-significant effects of the TA covariate (F1,157 = 2.35, *p* = 0.127, η^2^*p* = 0.015), Session (F1,157 = 3.30, *p* = 0.071, η^2^*p* = 0.021), and IES group (F1,157 < 1, *p* = 0.751, η^2^*p* < 0.01) were found. All the interactions of Session with Age, TA, or IES group were also non-significant (F1,157 = 1.70, *p* = 0.194, η^2^*p* = 0.01 for the interaction of Session with Age; F1,157 = 1.30, *p* = 0.255, η^2^*p* < 0.01 for the interaction of Session with TA; and F1,157 < 1, *p* = 0.603, η^2^*p* < 0.01 for the interaction of Session with IES group).

The follow-up ANOVA on the N-2 repetition cost showed no significant effects (F1,157 = 1.068, *p* = 0.303, η^2^*p* < 0.01, F1,157 < 1, *p* = 0.55, η^2^*p* < 0.01, F1,157 = 1.94, *p* = 0.166, η^2^*p* = 0.012, F1,157 < 1, *p* = 0.791, η^2^*p* < 0.01 for the main effects of IES Group, Session, Age, and TA, respectively; F1,157 < 1, *p* = 0.958, η^2^*p* < 0.01 for the interaction of Session with Age, F1,157 < 1, *p* = 0.638, η^2^*p* < 0.01 for the interaction of Session with TA; and F1,157 < 1, *p* = 0.791, η^2^*p* < 0.01 for the interaction of Session with IES group).

**Table A1:** Linear Mixed Models for lockdown effects on BART and IGT decision-making tasks.

	BART Adj. Avg. Pumps			IGT Net Score		
	*β*	*CI*	*p*	*β*	*CI*	*p*
			(*p boot*)			(*p boot*)
Intercept	−0.08	−0.22–0.05	0.234	−0.04	−0.12–0.05	0.435
			(0.114)			(0.116)
Week [linear]	−0.44	−0.88–−0.00	**0.049**	−0.12	−0.41–0.18	0.44
			(**<0.01**)			(**0.039**)
Week [quadratic]	−0.12	−0.29–0.05	0.168	−0.02	−0.14–0.09	0.723
			(0.424)			(0.762)
Week [cubic]	0.09	−0.03–0.22	0.154	0.04	−0.04–0.13	0.312
			(0.395)			(0.369)
Week [4th degree]	−0.01	−0.14–0.12	0.906	−0.04	−0.13–0.05	0.392
			(0.938)			(0.379)
Week [5th degree]	0.13	−0.03–0.30	0.117	−0.02	−0.13–0.09	0.73
			(0.309)			(0.724)
Previous sessions	0.32	0.16–0.47	**<0.001**	0.15	0.05–0.25	**0.004**
			(**<0.001**)			(**<0.001**)
Block [linear]	--	--	--	0.24	0.20–0.29	**<0.001**
						(**<0.001**)
Block [quadratic]	--	--	--	−0.04	−0.09–0.00	0.078
						(0.164)
Block [cubic]	--	--	--	−0.02	−0.07–0.02	0.342
						(0.428)
Block [4th degree]	--	--	--	0.02	−0.03–0.06	0.492
						(0.518)
Age	--	--	--	−0.09	−0.15–−0.04	**<0.001**
						(**<0.001**)

Note: BART = Balloon Analogue Risk Task, IGT = Iowa Gambling Task, *β* = beta estimates, *CI* = 95% confidence intervals, *p* = significance level, *(p boot)* = bootstrapped *p*-values. Bold values were statistically significant (*alpha* = 0.05).

**Table A2:** Linear Mixed Models for lockdown effects on CST task.

	CST Switch Cost			CST N-2 Rep. Cost		
	*β*	*SE*	*p*	*β*	*SE*	*p*
			(*p boot*)			(*p boot*)
Intercept	0.06	−0.08–0.20	0.417	0.1	−0.04–0.23	0.154
			(0.259)			(00.077)
Week [linear]	0.11	−0.37–0.59	0.656	0.43	−0.05–0.92	0.077
			(0.414)			(**<0.01**)
Week [quadratic]	0.08	−0.13–0.29	0.451	0.04	−0.24–0.31	0.798
			(0.577)			(00.804)
Week [cubic]	−0.22	−0.38–−0.07	**0.005**	0.07	−0.14–0.28	0.497
			(**0.044**)			(00.502)
Week [4th degree]	0.03	−0.13–0.19	0.693	0.03	−0.17–0.23	0.744
			(0.724)			(00.724)
Week [5th degree]	0.13	−0.08–0.34	0.217	0.01	−0.26–0.27	0.956
			(0.32)			(00.956)
Previous sessions	−0.26	−0.43–−0.10	**0.002**	−0.21	−0.38–−0.04	**0.016**
			(**<0.001**)			(**<0.001**)
Age	0.13	0.05–0.21	**0.001**	--	--	--
			(**<0.001**)			

Note: CST = Category Switch Task, *β* = beta estimates, *CI* = 95% confidence intervals, *p* = significance level, *(p boot)* = bootstrapped *p*-values. Bold values were statistically significant (*alpha* = 0.05).
